# Mnemonic factors associated with the tip-of-the-tongue phenomenon

**DOI:** 10.1038/s41598-025-96497-3

**Published:** 2025-04-24

**Authors:** James Christopher Barry, Emilio Ferrer, Garikoitz Lerma-Usabiaga, Pedro M. Paz-Alonso

**Affiliations:** 1 Basque Center on Cognition, Brain and Language (BCBL), Donostia-San Sebastián, Spain; 2 Department of Psychology, University of California, Davis, USA; 3 Ikerbasque, Basque Foundation for Science, Bilbao, Spain

**Keywords:** Tip-of-the-tongue, Memory, Lexical retrieval, Age-of-acquisition, Frequency of retrieval, Recency of retrieval, Human behaviour, Cognitive control, Language, Consolidation, Forgetting, Long-term memory

## Abstract

The tip-of-the-tongue (ToT) phenomenon is a transient semantic memory retrieval failure. Here we examined to what extent different mnemonic factors (i.e., age of acquisition, frequency of retrieval, recency of last retrieval) impact ToTs during the retrieval of famous faces and places. Eighty adults completed a self-paced experiment for both stimuli. This required making judgements on whether they knew the name, were in a ToT state, the image was familiar or the name was unknown, as well as completing follow-up questions examining the mnemonic factors of interest. Results revealed that later acquired names, a lower frequency of retrieval, and less recently encountered names, all predicted an increase in ToT occurrences. These findings followed a similar pattern across faces and places, with places being stronger predictors for each mnemonic factor. By examining these factors simultaneously across these semantic categories, we provide further evidence regarding the variables determining transient retrieval failures.

## Introduction

The tip-of-the-tongue (ToT) phenomenon is a transient retrieval failure of a word that is certain to be known. During this state, individuals may be able to recall the target word’s phonological or semantic features but cannot successfully retrieve the label. For decades, numerous studies have employed various paradigms and stimuli to examine how different factors can lead to an increase in ToTs. The present study aims to understand the impact of three mnemonic factors (i.e., age of acquisition, frequency of use, and recency of use) on ToT rates of semantically known faces and places. While there is some initial evidence about the role of these factors, the differing methodologies and stimuli previously used have led to inconsistent results regarding the extent to which these factors influence ToTs involving proper nouns, with no study investigating these factors together.

The predominant hypotheses concerning ToTs suggest they are caused by either phonologically related names “blocking” the intended target (Blocking Hypothesis^1^), or the target name activates the semantic representation but not the required phonological representations due to weakened inter-node connections (Transmission Deficit Hypothesis [TDH]^2^). While the blocking hypothesis and the TDH both suggest that ToTs result from insufficient direct access to the label, further phenomenological theories propose that ToTs are the result of metacognitive processes. For instance, the cue familiarity hypothesis (CFH)^3,4^ suggests that ToTs occur due to the degree to which a particular cue or question is recognised. Furthermore, ToTs may be the result of metacognitive processes which accumulate information about the target and when it reaches a specific criterion, a ToT is produced (for a review see^5^). Herein, we propose that mnemonic factors associated with the name (i.e., age of acquisition, frequency of retrieval, and recency of last retrieval) are the main determinants influencing the number of ToTs. To our knowledge, no previous study has: (1) examined these three mnemonic factors together in the same experiment and (2) extended these analyses to multiple semantic categories, i.e., famous faces and places.

One of the more investigated factors of lexical retrieval is the age of acquisition (AoA), which concerns the age when a name has been initially learned. Bonin and colleagues^6^ found a significant positive correlation between AoA and ToT rates of French celebrities, demonstrating that famous names acquired later in life are associated with an increase in ToTs. Furthermore, earlier acquired names may have been encountered more frequently and consolidated, strengthening the neural connections and minimising ToT occurrences^7^. While AoA effects have previously been examined with famous faces, no study has examined to what extent the AoA of famous places affects ToT occurrences.

Intuitively, the frequency at which a name is retrieved is linked to AoA, because names acquired earlier may have more opportunities for retrieval. While there is a high correlation between frequency and AoA, these factors may influence retrieval attempts independently^8^. Although the frequency of common nouns is established by how often that word appears in a corpus, measuring the frequency of proper nouns can be problematic. Considering this, most ToT studies exploring frequency effects used diary entries [e.g. ^2,9^] and ascertained that ToTs were most common for highly familiar friends and family. This may be due to the increased opportunity for a ToT to occur due to more frequent retrieval^10^. This is in contrast to common nouns where low-frequency words have been shown to elicit more ToTs due to weakened connections between the lexical, phonological and semantic properties of the word^2,11^. While it has been demonstrated that frequently used names lead to more ToTs, the distance from when the name was last retrieved may also be influential.

Instinctively, names with a greater interval since their last retrieval may lead to more ToTs, compared to names with a shorter interval. Rastle and Burke^12^ examined this effect with common nouns by exposing participants to half the words in an unrelated task before a ToT elicitation session, discovering that ToT occurrences were significantly reduced for the words primed in a previous session. While also finding that proper nouns produced more ToTs, they found no evidence that this was related to the recency of use.

Cleary and colleagues examined the recency effect on famous faces^13^ and famous places^14^ by having participants study half the items immediately before a ToT elicitation task. They discovered that, for famous faces, no differences in the number of ToTs were observed between names that were studied prior and unstudied names. However, for famous places, fewer ToTs were reported for names that were studied compared to unstudied names. In summary, for famous places, ToT occurrences were significantly reduced for names encountered more recently, compared to names encountered less recently. However, this effect was not observed for famous faces.

A caveat to these studies examining recency effects is that the names were primed immediately before the testing session. Therefore, these studies cannot accurately represent the distances that occur naturally throughout life. ToTs may increase with age^15^ due to the greater interval since the name was last retrieved, which inevitably increases in distance as people age. While this effect has been observed in different age groups^2^, to our knowledge, no study has experimentally examined the effect of recency of famous name retrieval in a homogenous age group without using immediate priming. As with frequency, it is thought that the increased time from when a name was last retrieved weakens the neural connections, causing an increase in ToTs.

While efforts have been made to understand the neurobiological basis of ToTs ^[[e.g. 16,17]]^, these studies used general knowledge questions or famous faces to elicit ToTs, while predominantly examining the effect of age. These studies examining ToTs have uncovered activations in the anterior cingulate cortex (ACC) and the dorsolateral prefrontal cortex (dlPFC) due to these regions’ involvement in conflict detection^18^ and resolution^19^, respectively. Furthermore, evidence from arousal-based pupil dilation during ToTs suggests that ToTs involve metacognitive processes including curiosity and information seeking^20^. Ryals and colleagues further suggested that the increased pupil dilation during ToTs may reflect an increased amount of access and retrieval of partial information about the target. However, it is unknown to what extent mnemonic factors, such as frequency or recency, have on the neurobiological mechanisms of ToTs and whether this extends across multiple semantic categories.

Given previously mixed findings regarding the factors that can lead to ToTs, our aims are: (1) to examine the AoA, frequency and recency factors simultaneously within the same study to investigate their influence on ToTs, and (2) to examine whether the impact of these factors is consistent across famous faces and places. It is expected that: (1) names with a later AoA will precipitate an increase in ToTs due to the reduced possibilities of consolidation^21^; (2) names that are retrieved less frequently and which have a greater interval of last retrieval will lead to an increased ToT occurrence due to the weakening of connections between the lexical, phonological and semantic representations^2^; (3) no statistical differences will be observed between famous faces and places for the AoA and frequency factors. However, it is expected that differences between the stimuli will be observed for the recency factor, in that famous places will lead to more ToTs^14^.

## Results

### Distribution of responses

**Fig. 1:**
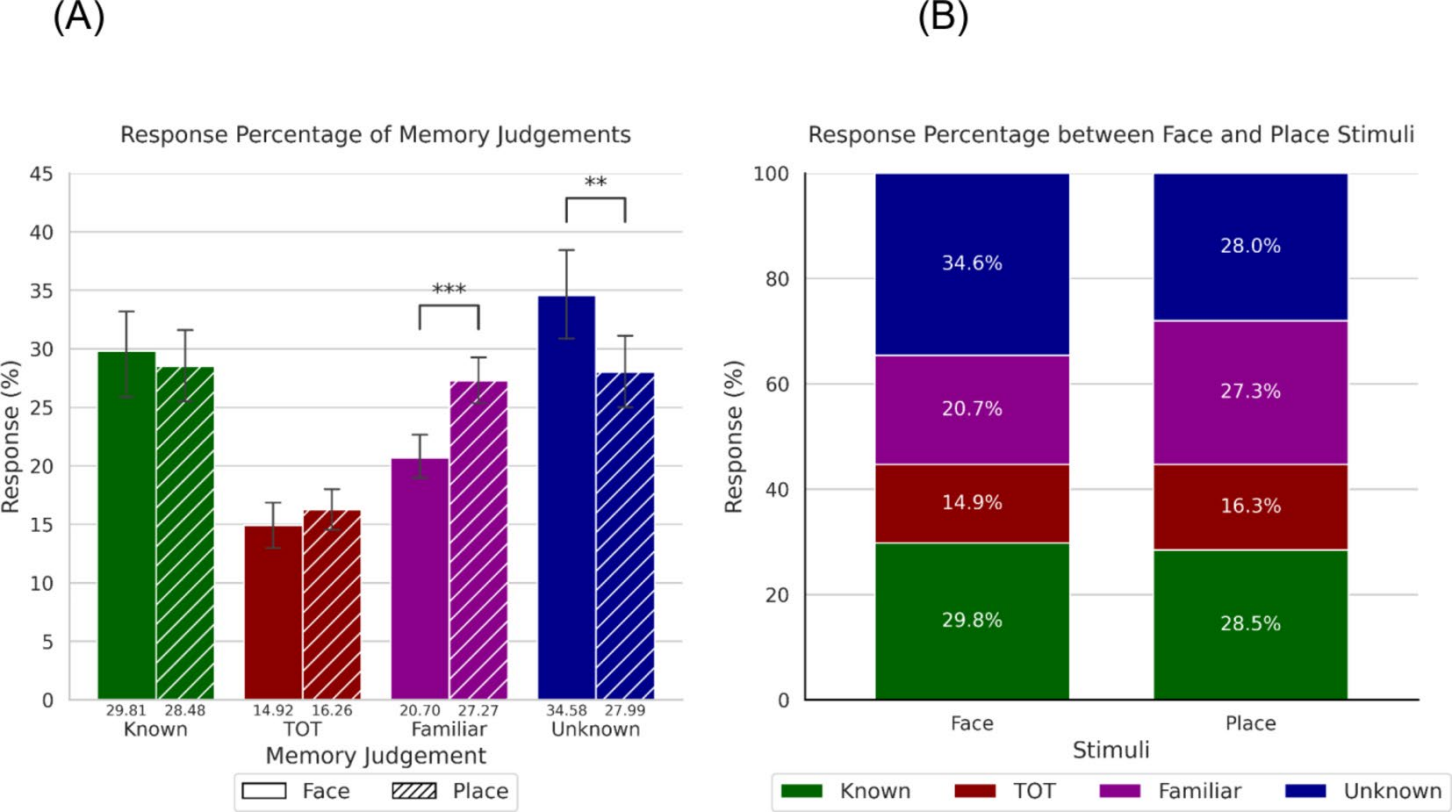
Overall percentages of memory judgements. Panel** A** represents the percentage of responses as a function of memory judgement and stimuli. Panel** B** represents the cumulative percentage of memory judgement between each stimuli. Error bars represent 95% confidence intervals.

Figure 1 represents the overall percentage of responses for each of the four types of mnemonic alternatives separated for faces and places. A 2 (Stimuli: face, place) x 4 (Memory Judgement: known, ToT, familiar, unknown) repeated measures ANOVA was conducted to explore statistical differences between the stimuli and mnemonic alternatives, as well as their interaction. This analysis revealed a statistically significant main effect of Memory Judgement, *p* = < 0.001, n_*p*_^2^ = 0.299, that was qualified by a statistically significant Memory Judgement by Stimuli interaction, *p* = < 0.001, n_*p*_^2^ = 0.103. The main effect of Stimuli was not statistically significant, (*p* = .619, n_*p*_^2^ = 0.004). When exploring the interaction between Stimuli within each Memory Judgement, simple effect post-hoc analysis revealed a higher percentage of responses for places than faces in the familiar condition, *p* = < 0.001, *d* = 0.764, as well as a higher percentage of responses for faces relative to places in the unknown condition, *p* = .007, *d* = 0.428. No statistically significant differences were observed between faces and places for the known (*p* = .634, *d* = 0.065) and ToT (*p* = .168, *d* = 0.166) conditions.

### Responses to individual questions

**Fig. 2:**
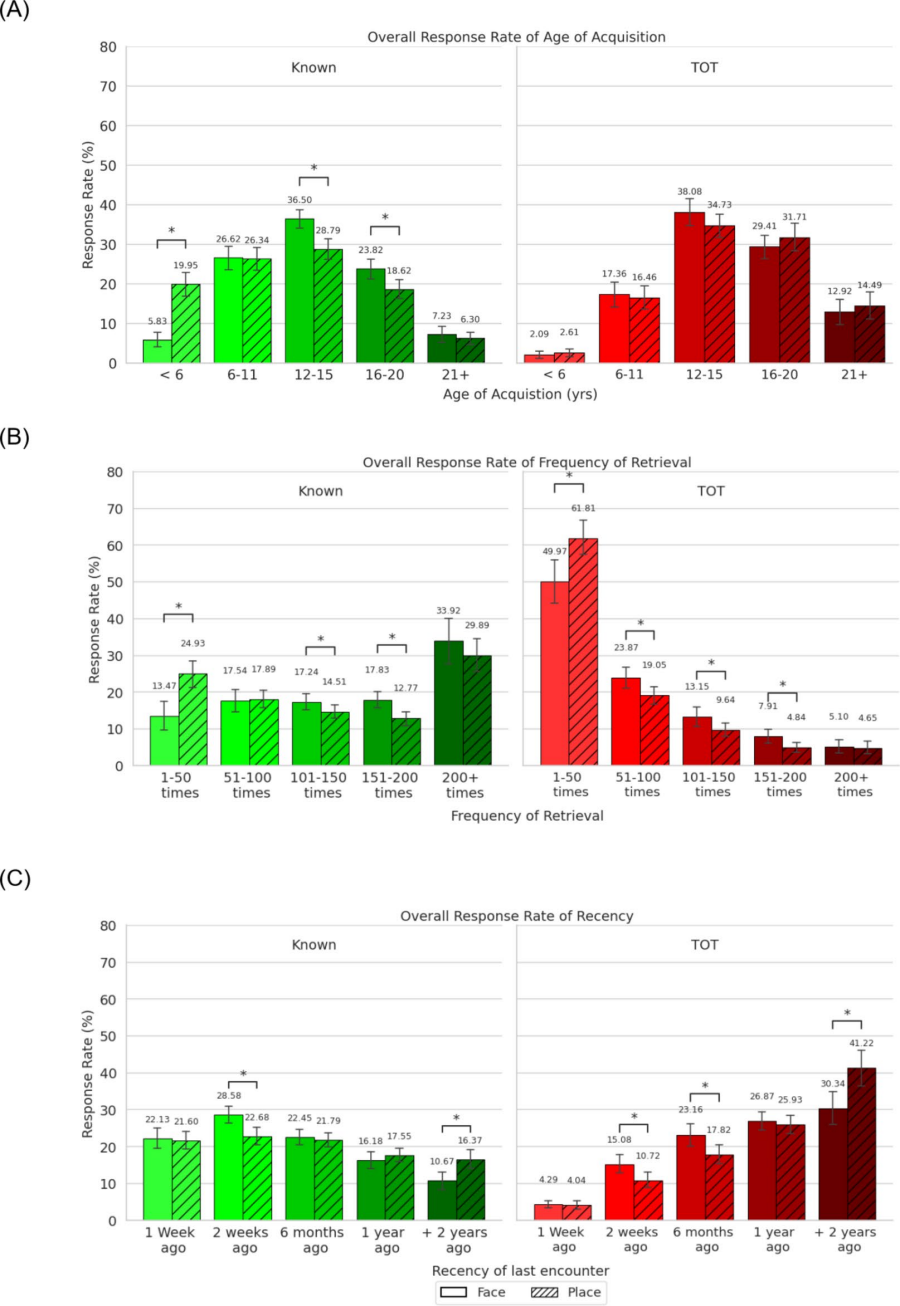
Percent of responses to each of the three questions (i.e Age of acquisition (**A**), frequency (**B**) and recency (**C**) separated by Known (left) and ToT responses (right), as well as by stimuli within each plot. error bars represent 95% confidence interval. ^*^p ≤ .05.

To examine if there was an interaction between the Judgement, Stimuli and Question Responses, a series of 2 (Judgement: known, ToT) x 2 (Stimuli: face, place) x 5 (Question Response: the individual responses to the questions) repeated measures ANOVAs were conducted (total of 3 ANOVAs). A statistically significant Judgement x Stimuli x Question Response interaction was observed for each of the three question types (*ps* ≤ 0.001). To better understand the known and ToT judgements individually, a series of 2 (Stimuli: face, place) x 5 (Question Response: the individual responses for each question; see Fig. 2) repeated measures ANOVAs were conducted on each question within either the known or ToT responses separately (total of 6 ANOVAs) to examine statistical differences between the Stimuli and the Question Responses, as well as their interaction.

### Age of acquisition

The ANOVA for the known judgements revealed a statistically significant main effect of Question Response, *p* = < 0.001, n_*p*_^2^ = 0.536, which was further qualified by a statistically significant Stimuli by Question Response interaction, p = < 0.001, np2 = 0.678. Simple-effect post-hoc analysis of this interactive effect showed a higher percentage of known responses for faces than places in the “12–15 years” condition, *p* = < 0.001, *d* = 0.820, as well as in the “16–20 years” condition, *p* = < 0.001, *d* = 0.637. The post-hoc analysis also revealed a higher percentage of responses for places relative to faces in the “< 6 years” condition, *p* = < 0.001, *d* = 1.465. No statistically significant differences were observed between faces and places for the “21 + years” (*p* = .073, *d* = 0.201) and the “6–11 years” (*p* = .457, *d* = 0.094) conditions.

The ANOVA for the ToT judgements revealed statistically significant main effects of Stimuli, *p* = .007, n_*p*_^2^ = .110, and Question Response, *p* = < .001, n_*p*_^2^ = .584. However, the Stimuli by Question Response interaction was not statistically significant (*p* = .201, n_*p*_^2^ = .023). Simple effects post-hoc analysis of the main effect of Question Response demonstrated that there was a statistically significant higher number of responses for the “6–11 years” condition compared to the “< 6 years” condition, *p* = < .001, *d* = 1.848. Furthermore, there were a statistically significant higher number of responses for the “12–15 years” condition compared to the “< 6 years”, *p* = < .001, *d* = 3.936, “6–11 years”, *p* = < .001, *d* = 1.66, “16–20 years”, *p* = .005, *d* = .539, and “21 + years”, *p* = < .001, *d* = 1.82, conditions. There was also a statistically significant higher number of responses for the “16–20 years’’ condition compared to the “< 6 years”, *p* = < .001, *d* = 3.286, “6–11 years”, *p* = <0.001, *d* = 1.13, and “21 + years” conditions, *p* = < 0.001, *d* = 1.34. Finally, a statistically significant higher number of responses was observed for the “21 + years” condition compared to the “< 6 years” condition, *p* = < 0.001, *d* = 1.06. No statistically significant differences were observed between the “21 + years” and “6–11 years” conditions (*p* = .076, *d* = 0.351).

### Frequency

Both ANOVAs for the known, *p* = < 0.001, n_*p*_^2^ = 0.271, and ToT, *p* = < 0.001, n_*p*_^2^ = 0.743, judgements identified a statistically significant main effect of Question Response. For both the known, *p* = < 0.001, n_*p*_^2^ = 0.171, and ToT, *p* = < 0.001, n_*p*_^2^ = 0.221, judgements, this main effect was qualified by a statistically significant Stimuli by Question Response interaction. No statistically significant main effect of Stimuli was observed for either the known (*p* = .394, n_*p*_^2^ = 0.013) or ToT (*p* = .133, n_*p*_^2^ = 0.037) judgements.

For the interaction between Stimuli and Question Response for the known judgement, simple effect post-hoc analysis revealed a higher percentage of responses for faces relative to places for both the “101–150 times” condition, *p* = < 0.031, *d* = 0.366, and the “151–200 times” condition, *p* = < 0.001, *d* = 0.714. Furthermore, this post-hoc analysis also revealed a higher percentage of responses to places compared to faces in the “1–50 times” condition, *p* = < 0.001, *d* = 0.822. No statistically significant differences were observed between faces and places for both the “200 + times” (*p* = .384, *d* = 0.105) and “51–100 times” (*p* = .885, *d* = 0.018) conditions.

An exploration into the interaction between Stimuli and Question Response for the ToT judgement revealed a higher percentage of responses for places than faces in the “1–50 times” condition, *p* = < 0.001, *d* = 0.623. However, this analysis also revealed a higher percentage of ToT responses for faces relative to places in the “51–100 times”, *p* = .008, *d* = 0.415, “101–150 times”, *p* = .002, *d* = 0.417, and “151–200 times”, *p* = < 0.001, *d* = 0.608, conditions. No statistically significant difference was observed between faces and places for the “200 + times” (*p* = .268, *d* = 0.150) condition.

### Recency

Repeated measures ANOVAs for the Recency question identified a statistically significant main effect of Question Response for both the known, *p* = < 0.001, n_*p*_^2^ = 0.187, and ToT, *p* = < 0.001, n_*p*_^2^ = 0.518, judgements. This was then further qualified by a statistically significant Question Response by Stimuli interaction for both the known, *p* = < 0.001, n_*p*_^2^ = 0.152, and ToT judgements, *p* = < 0.001, n_*p*_^2^ = 0.198. No statistically significant main effect of Stimuli was observed for either the known (*p* = .309, n_*p*_^2^ = 0.016) or ToT (*p* = .999, n_*p*_^2^ = 0) judgements.

The simple effect post-hoc analysis exploring the interaction between Stimuli and Question Response for the known judgement revealed a higher percentage of responses for faces compared to places in the “2 weeks ago” condition, *p* = < 0.001, *d* = 0.648. However, a higher percentage of responses were revealed for places compared to faces for the “+2 years ago” condition, *p* = < 0.001, *d* = 0.622. No statistically significant differences were observed between faces and places for the “1 week ago” (*p* = .832, *d* = 0.025), “6 months ago” (*p* = .43, *d =* 0.121) and “1 year ago” (*p* = .129, *d* = 0.167) conditions.

Exploring the interaction between Stimuli and Question Response for the ToT judgement revealed a higher percentage of responses for places compared to faces in the “+2 years ago” condition, *p* = < 0.001, *d* = 0.596. However, a higher percentage of responses were revealed for faces compared to places for the “2 weeks ago”, *p* = .002, *d* = 0.471, and “6 months ago”, *p* = < 0.001, *d* = 0.524, conditions. No statistically significant differences were observed between faces and places for the “1 week ago” (*p* = .538, *d* = 0.091) and “1 year ago” (*p* = .737, *d* = 0.053) conditions.

### Associations between age and memory judgements

**Fig. 3:**
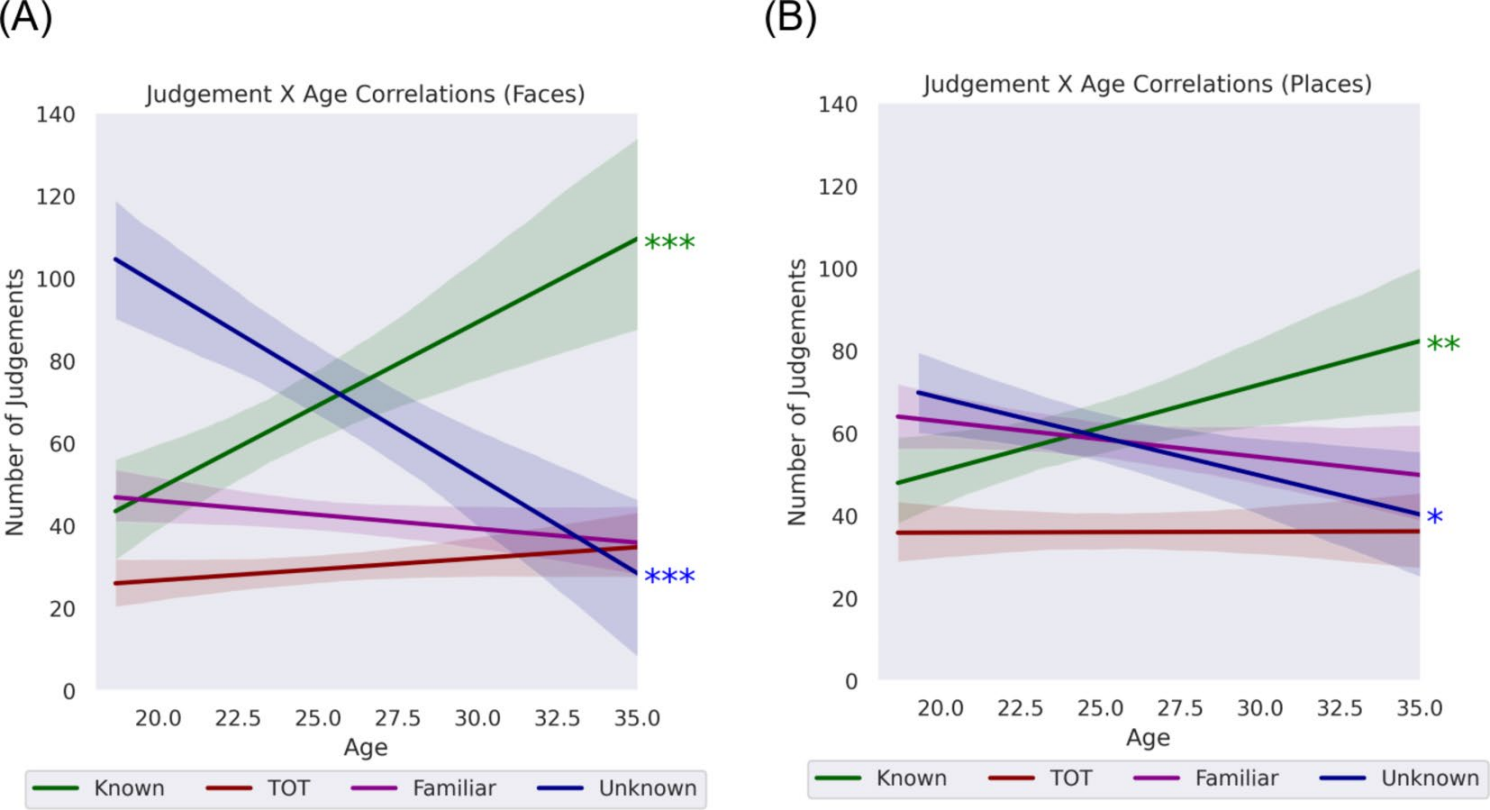
Pearson(*r*) correlations between age and memory judgements for both faces (**A**) and places (**B**).

Pearson correlations were conducted to examine associations between Age and Memory Judgements, as an increase in ToTs has been found to correlate with an increase in age, particularly after the age of 35^15^. Figure 3 represents these correlations separated for faces (A) and places (B). Both faces, *r*(79) = 0.434, *p* = < 0.001, and places, *r*(80) = 0.316, *p* = .004, exhibited significant positive associations between age and the known judgement. In contrast, significant negative correlations were also observed for both faces, *r*(80) = − 0.498, *p* = < 0.001, and places, *r*(78) = − 0.286, *p* = .011, between age and the unknown judgement. For both types of stimuli, no significant correlations were observed between age and ToTs nor familiarity. This is likely to be due to the participants’ age range of 18-to-35 years.

### Logistic regression results

A logistic regression was performed to examine the effects of each of the three questions (AoA, Frequency and Recency) on the likelihood of producing either a known or a ToT judgement. In this model, known was coded as “0”, and ToT judgements were coded as “1”.

**Fig. 4:**
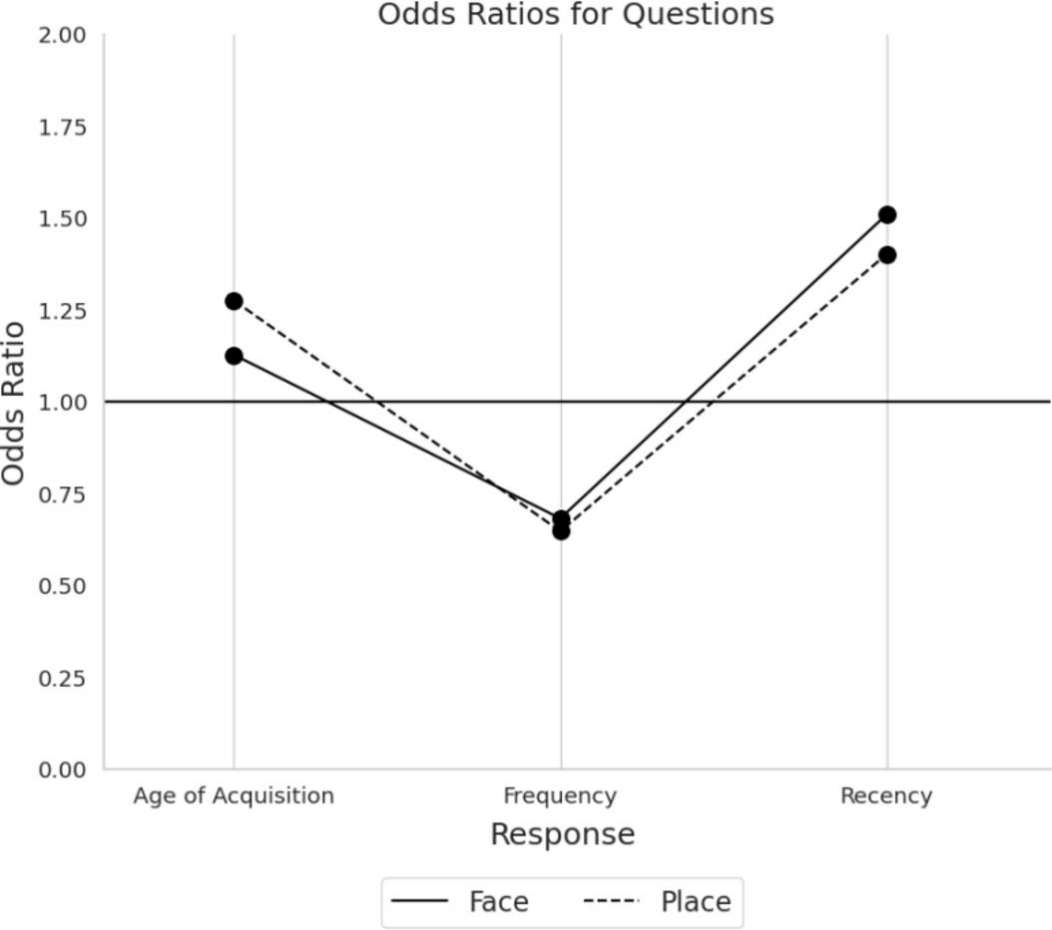
Line graph plotting the odds ratios for the questions taken as a whole. Values > 1 = increased ToT likelihood; Values < 1 = decreased ToT likelihood. For results of statistical analyses, see Table 1.

Table 1 shows the results of the logistic regression when grouping the answers together into their respective questions. Both face and place stimuli performed similarly across all predictors. The strongest predictor for a ToT judgement was Recency for both faces (*OR* = 1.508, 95% CI [1.459, 1.559], *p* = < 0.001) and places (*OR* = 1.456, 95% CI [1.4, 1.515], *p* = < 0.001). Furthermore, AoA was also a positive predictor for ToT judgements for both faces (*OR* = 1.126, 95% CI [1.079, 1.176], *p* = < 0.001) and places (*OR* = 1.274, 95% CI [1.218, 1.333], *p* = < 0.001), albeit, not as strong a predictor as Recency. Finally, Frequency proved to be a significant negative predictor for ToT judgements for both faces (*OR* = 0.681, 95% CI [0.661, 0.701], *p* = < 0.001) and places (*OR* = 0.672, 95% CI [0.646, 0.699], *p* = < 0.001). Overall, for both faces and places, AoA and Recency were both positive predictors of ToTs, while Frequency was a negative predictor in that the more frequently the name had been retrieved predicted a decrease in the number of ToT occurrences (See Fig. 4). However, given the variability of the distribution of the answers within each question, it was decided to run a logistic regression separately for each question, using the answers to each individual question as predictors.

**Table 1:** Summary of odds ratios for each of the predictors separated between face and place stimuli.

Predictor	Face stimuli			Place stimuli		
		CI			CI	
	OR	5%	95%	OR	5%	95%
Questions combined						
(Intercept)	0.512*	0.410	0.640	0.338*	0.265	0.431
Age of Acquisition	1.126*	1.079	1.176	1.274*	1.218	1.333
Frequency	0.681*	0.661	0.701	0.672*	0.646	0.699
Recency	1.508*	1.459	1.559	1.456*	1.400	1.515
Age of acquisition						
(Intercept)	0.884*	0.845	0.925	0.907*	0.871	0.945
< 6 years	0.408*	0.338	0.491	0.221*	0.193	0.253
6–11 years	0.770*	0.709	0.836	0.750*	0.670	0.816
12–15 years	1.221*	1.140	1.307	1.319*	1.225	1.421
16–20 years	1.411*	1.309	1.520	1.791*	1.650	1.945
Over 21 years	1.611*	1.455	1.783	2.316*	2.071	2.590
Frequency						
(Intercept)	0.914*	0.883	0.947	0.717*	0.686	0.748
1 to 50	2.616*	2.437	2.807	3.346*	3.112	3.567
51 to 100	1.514*	1.403	1.634	1.544*	1.414	1.687
101 to 150	0.969	0.889	1.056	1.010	0.908	1.123
151 to 200	0.699*	0.637	0.766	0.641*	0.566	0.727
Over 200	0.343*	0.315	0.373	0.214*	0.188	0.244
Recency						
(Intercept)	0.870*	0.840	0.901	0.793*	0.761	0.826
1 week ago	0.407*	0.364	0.456	0.211*	0.184	0.242
1 month ago	0.770*	0.711	0.834	0.560*	0.506	0.619
6 months ago	1.220*	1.135	1.312	1.034	0.948	1.127
1 year ago	1.411*	1.309	1.520	2.008*	1.842	2.188
Over 2 years ago	1.611*	1.487	1.746	3.232*	2.982	3.502

*CI* confidence interval, *OR* Odds ratio, *p < .05.

### Age of acquisition

**Fig. 5:**
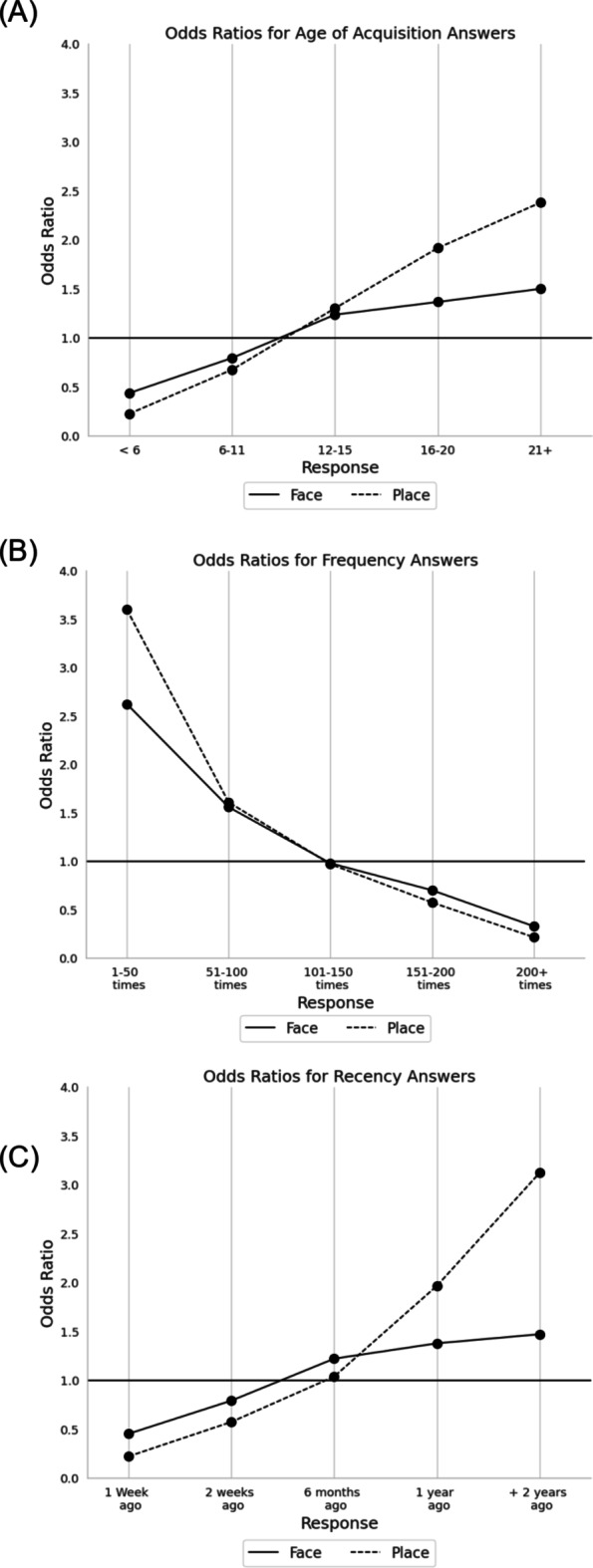
Line graphs plotting the odds ratios for the answers to each mnemonic question (i.e. age of acquisition (**A**), Frequency (**B**) and Recency (**C**). Values > 1 = increased ToT likelihood; Values < 1 = decreased ToT likelihood; For results of statistical analyses, see Table 1.

Both face and place names produced significant results on the answers to the question involving what age the name of the image was first acquired (see Table 1), as well as performing similarly in that, the longer it has been since the name was first acquired, the less likely it is to produce a ToT. When comparing across stimuli, place names (*OR* = 1.791, 95% CI [1.65, 1.945], *p* = < 0.001) acquired between the ages of 16–20 years were stronger predictors of ToTs when compared to face names acquired during the same age period (*OR* = 1.411, 95% CI [1.309, 1.52], *p* = < 0.001). This effect was more pronounced for names acquired after the age of 21, where place names (*OR* = 2.071, 95% CI [2.071, 2.59, *p* = < 0.001) were a much stronger predictor of producing a ToT compared to face names (*OR* = 1.611, 95% CI [1.455,1.783], *p* = < 0.001) acquired after the age of 21. Overall, these results suggest that names encountered for the first time more recently have a greater chance of producing a ToT (See Fig. 5).

### Frequency

The second question of the study explored how often the name of the face or place has been retrieved over the lifespan. Both face and place names performed similarly in that names that were retrieved less than 100 times better predicted ToTs (see Table 1). Likewise, face and place names that were retrieved more than 151 times were better predictors of known judgements. Names retrieved between 101 and 150 times for both faces (*p* = .472) and places (*p* = .566) were not statistically significant and predicted neither ToT nor known judgements. Between stimuli, place names that were retrieved 1 to 50 times (*OR* = 3.346, 95% CI [3.112, 3.567], *p* = <0.001) were much higher predictors of ToT judgements compared to face names of the same frequency (*OR* = 2.616, 95% CI [2.437, 2.807], p = < 0.001). In summary, this logistic regression suggests that, for both face and place names, ToTs are more likely to occur when the name is retrieved less over the lifespan, with low frequency of retrieval for place names being a much higher predictor of a ToT experience (See Fig. 5).

### Recency

Overall, the results for both face and place names performed similarly in that names encountered less than 1 month ago were less likely to produce a ToT experience, while names encountered more than 1 year ago were more likely to produce a ToT response (see Table 1). However, names encountered 6 months ago for both faces (*p* = .093) and places (*p* = .412) failed to predict either a known or a ToT response. Between faces and places, place names last encountered over two years ago (*OR* = 3.232, 95% CI [2.982, 3.502], *p* = < 0.001) were a much stronger predictor of a ToT response when compared with face names of the same length of time (*OR* = 1.611, 95% CI [1.487, 1.746], *p* = < 0.001). Taken together, these results suggest that the greater the length of time from when a name was last encountered has a higher chance of causing a ToT experience, with place names being greater predictors compared to face names (See Fig. 5).

## Discussion

In this study, we aimed to clarify to what extent AoA, frequency, and recency of use of known faces and places influence ToT occurrences. Results revealed that later acquired names, a lower retrieval frequency, and less recently encountered names, predicted an increase in ToTs. These findings demonstrated a similar pattern across faces and places, with places being stronger predictors for each mnemonic factor. These main results are discussed next.

A later AoA of famous faces and places were related to an increase in ToTs. While this effect has been observed with common nouns^22^ and famous faces^6^, we extended it to famous places, which have not been previously utilised to investigate how AoA influences ToTs. These results further highlight the nature of memory consolidation, specifically that first-learned words are the last to be forgotten. Built upon the semantic hypothesis^23^, Steyvers and Tenenbaum^24^ created a model that suggested earlier learned names are semantically stronger due to forming a denser network of connections of related information, ensuring more automatic and effortless retrieval of earlier learned names. Conversely, more recently learned names have had less opportunity to create a dense semantic network, resulting in more retrieval errors. Given the linear nature of our results, the earlier acquired names of faces and places may have had more opportunities to build a dense semantic network. This could also account for the observed differences, with place names having more chances to produce ToTs than face names when acquired more recently, due to famous place names having less opportunity to build a dense network compared to famous face names, which are typically more prevalent in mass media.

However, this hypothesis does not sufficiently account for frequency effects, because an earlier AoA may involve more frequent retrieval. Considering these two factors naturally interact, separating them has proven difficult^8^. By collapsing the answers to each mnemonic question together, our findings demonstrated that, compared to frequency effects, the AoA factor is a larger predictor of ToTs. While AoA may influence the semantic connections between the label and related information, more frequent retrieval may strengthen the connection to the label.

Regarding frequency, our results showed that faces and places retrieved less often produced more ToTs. Bonin and colleagues^6^ found that less frequently retrieved famous faces were correlated with an increase in ToTs. However, previous ToT studies using diary entries^2,9^ found that proper nouns involving highly familiar friends and family were associated with more ToTs due to increased possibilities for a ToT to occur. Although this result may contradict our findings, this may be due to the nature of the retrieved name. The previously mentioned diary studies reported an increase in ToTs for personally known friends and family, while our results report ToTs related to celebrities with whom our participants presumably do not interact directly. More studies are needed to understand the differences underlying the retrieval of personally known and famous names, interactions in this regard between episodic and semantic memories^25^, and potential changes in these differences over development [e.g. ^26^].

Our findings regarding frequency of retrieval support the TDH^2^ in that less frequent use can cause the connections within the relevant networks to weaken. However, while the TDH suggests that weakened connections between the semantic and phonological nodes cause a ToT, we argue that a weakened connection between the recognition of the face or place and the retrieval of the label is responsible. Frequent retrieval of a name strengthens the connections between the networks supporting recognition and retrieval, resulting in successful access to the label. However, less frequent retrieval may cause connections within and between these networks to weaken, resulting in failed access to the label and causing a ToT.

Furthermore, these findings support the cue-familiarity hypothesis, in that names that have been retrieved more frequently lead to stronger associations between the name and its related cues, thereby reducing the number of occurrences ToT experiences. This may also explain the increase in ToTs when a name is retrieved less frequently, as the associations between the name and the related cues are weak. This triggers a reduced sense of familiarity with the cue, resulting in a ToT experience.

This weakening of the connections, as well as the reduced association between the name and the cue, may also be caused by less recent use which we also found predicted more ToTs across famous faces and places. Similar to AoA, we found a linear gradient in that more recently retrieved names were less likely to predict a ToT due to the strengthening of connections. This finding is consistent with the testing effect^27^ which posits that memories are strengthened through active retrieval, with more effortful retrieval resulting in stronger memories^28^. This effect has been shown to strengthen the associations between the label and related information^29^ and improve the ability to inhibit competitors^30^. Therefore, the testing effect may help to explain the reduction in ToT occurrences through more recent retrieval of the labels. However, further studies are needed to examine the direct interaction between the testing effect and ToTs across different semantic categories.

A potential limitation to examining Age of Acquisition, Frequency, and Recency effects is whether ToTs influence subsequent mnemonic evaluations. Despite the participants being trained to understand the questions and responses before the beginning of the experiment, it might be possible that ToT experiences can affect later metamemory judgements, potentially biasing the results. To our knowledge no studies have examined the interactions between ToTs and subsequent mnemonic evaluations and, therefore, more studies are needed to examine whether this effect exists.

While the fusiform face area^31^ and the parahippocampal place area^32^ are regions specialised in recognising faces and places, respectively, it is thought that the left anterior temporal lobe (ATL) acts as a semantic storage hub^33^. Less frequent and less recent retrieval for both face and place names may weaken the connections within and between these regions, causing the inability to retrieve the correct label and resulting in a ToT. According to the posterior medial (PM) anterior temporal (AT) framework^34^, place name retrieval is processed within a PM network, while face name retrieval is processed within an anterior temporal (AT) network^35^, with multiple integration regions connecting both networks. Although the PM/AT networks do not include the predominant regions implicated in cognitive control (e.g. dlPFC, ACC), there may be interactions between these control regions and the PM/AT networks during transient semantic retrieval failures. Given the different regions and networks responsible for face and place name retrieval, future neuroimaging studies could investigate the PM-AT network across different stimuli during successful and unsuccessful recall to uncover regions responsible for the retrieval failure, and whether these regions are affected by frequency and/or recency of retrieval.

A consistent finding across all three mnemonic factors was that famous place names were better predictors of ToTs than famous face names. While Cleary and colleagues found incidental differences between face and place stimuli across two separate studies^13,14^, to our knowledge, no study has directly examined differences between faces and places within the same study across multiple mnemonic factors. With social media and streaming platforms becoming easier to access, it is more likely to encounter famous face names than place names, which can provide differential opportunities to create dense semantic networks related to these different categories. Furthermore, given that social media usage among young adults has surpassed traditional media usage^36^, famous face names can be encountered more frequently and recently, strengthening connections to the label. Future studies examining the neural networks and brain regions responsible for face and place retrieval (e.g. the posterior-medial anterior-temporal model^34^) may be able to identify differences between ToTs across different semantic categories.

By examining three mnemonic factors related to the to-be-retrieved label (i.e. AoA, frequency and recency of retrieval) across two proper noun categories (famous faces and places), we provide further evidence to better understand the mechanisms underlying ToTs. We propose that ToTs arise from weakened connections within and between specific brain networks responsible for recognition and naming and that these weakened connections can be influenced by the mnemonic factors examined in the present study.

## Methods

### Participants

Eighty young adult participants (age M = 25.60±,5.83 years, 44 females) were recruited. All participants had normal or corrected-to-normal vision and no history of neurological or psychiatric disorders. The presence of local famous faces and places used in the stimuli meant participants were required to be either local or lived in the area for a minimum continuous period of five years. Before taking part in the experiment, all participants gave written informed consent in compliance with the ethical regulations established by the Basque Center on Cognition, Brain and Language’s Ethics Committee and the guidelines of the Helsinki Declaration. Participants received monetary compensation for their involvement. A *post hoc* power analysis using G*Power (Version 3.1.9.2) revealed that the sample size (*n* = 80) provided a power of 79% to detect a medium effect size (*OR* = 2) with a two-tailed alpha value of 0.05^37,38^.

### Stimuli

Stimuli consisted of 220 images of famous faces and 220 images of famous places. Stimuli selected for the task had a recognition rate of at least 20% from a previously run normative study in the Basque Country with a population representative of the current study. Both face and place stimuli were balanced for their level of origin (local, national, international). However, race/ethnicity was not balanced due to approximately two-thirds of the faces being nationally (Spain) or locally (Basque Country) famous. Face stimuli were cropped to feature a clearly visible close-up view with nothing obscuring the face that is not typical of their appearance and approximately balanced for gender and profession (e.g., politician, actor, chef). The place stimuli did not contain anything in the image that would distract from the place, for example, people, and were approximately balanced for the category of the type of place (e.g., landmark, plaza, bridge). Additionally, all names of places were presented in the manner that a local would perceive it, rather than the international name (e.g., “La Casa Blanca” as opposed to “The White House”).

### Procedure

The experiment followed a self-paced design and consisted of two counterbalanced sections with identical instructions: one involving the face stimuli and the other the place stimuli. Following the instructions, all participants completed an example of the entire procedure. All participants completed both sections.

**Fig. 6:**
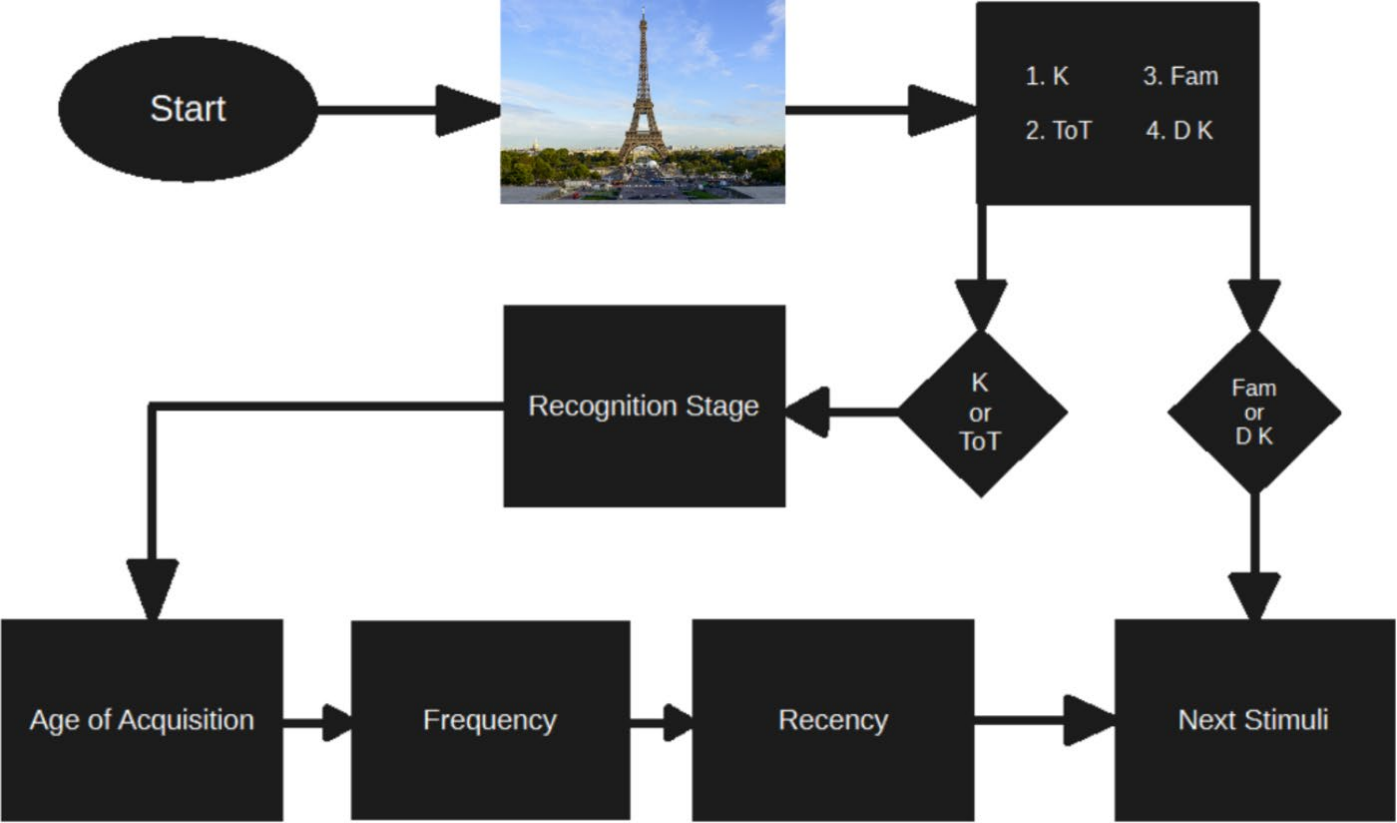
Depiction of the Experimental Procedure. Participants were presented with a famous face or place image and must respond with “Known”, “ToT” (tip-of-the-tongue), “Familiar”, or “Unknown”. If participants respond with “Familiar” or “Unknown”, another image is shown and the task continues. If participants respond with “Known” or “ToT”, a four-alternative recognition stage is presented which includes the correct name (Eiffel Tower), as well as three structurally similar distractors (i.e. Perret Tower, Pleyel Tower, Egée Tower). Following the recognition stage, the three questions (i.e. Age of Acquisition, Frequency and Recency) are presented. The possible choices for the Age of Acquisition question are less than 6 years, 6–11 years, 12–15 years, 16–20 years and 21 + years. The response options for the Frequency question include 1–50 times, 51–100 times, 101–150 times, 151–200 times and more than 200 times. The response options for the Recency question include a week, a month, 6 months, 1 year and 2 + years ago. Each option within each stage is given a number and the participant is required to press the corresponding number on a keyboard which represents their choice. After these four follow-up questions, the task continues with the next stimuli. *Note*: Each stage in this figure following the initial memory judgement choice is a representation of what the participant views.* K * Known,* ToT*  Tip-of-the-tongue,* Fam* Familiar,*DK * Don´t Know. Source of Eiffel Tower image: pixabay.com (https://pixabay.com/photos/paris-eiffel-tower-france-4563750/).

First, participants viewed an image of a famous face or place followed by a decision task asking the participants whether they could recall the name of the image (“known”), experienced a tip-of-the-tongue (“ToT”), recognised the image but do not know the name or are far from accessing it (“familiar”), or did not know the image (“don’t know”; see Fig. 6). ToTs were defined as “when the participant is unable to think of the word but feels sure that they know it and it is on the verge of coming to them” (Brown and McNeill, 1996). On the other hand, a familiar judgement is when the person or place seems familiar but the participant does not know the name. If the participant answered “known” or “ToT” in the first question, they were then presented with a four-alternative recognition task where they were required to select the correct name. The recognition task was presented to both confirm the known judgement and to validate the ToT judgement. The distractors in the recognition task were invented and were structurally and linguistically similar to the target. Following this recognition part, three follow-up questions (i.e., age of acquisition, frequency and recency) with a selection of five choices were presented. The first question involved recalling around what age the name of the image was first encountered or the *age of acquisition*, with the response options representing different stages of life (e.g., primary school, secondary school, etc.). The second question asked how many times the name had been retrieved over their lifetime or *frequency*: 1–50 times, 51–100 times, 101–150 times, 151–200 times and more than 200 times. The final question asked when the name of the image was last encountered or *recency*: a week, a month, 6 months, 1 year and 2 + years ago.

### Analysis

In total, three different sets of analyses were performed. First, distributional analyses of both the mnemonic responses and the answers to the questions that involved AoA, Frequency and Recency were performed. Outliers of 2.5sd ± away from the mean were removed. A 2 (Stimuli: face, place) x 4 (Memory Judgement: known, ToT, familiar, unknown) repeated measures ANOVA was conducted on the mnemonic response, while a 2 (Stimuli) x 5 (Question Response) repeated measures ANOVA was conducted on each follow-up question separated between known and ToT judgements. Second, to confirm no correlation between age and ToT judgements existed that may need to be subsequently controlled for, Pearson r correlations were performed separately for both face and place stimuli to examine the association between age and memory judgement. An alpha level of 0.05 was used for all tests and effect sizes are reported as n_*p*_^2^ for the ANOVAS and Cohen’s *d*s for the *t-tests*.

Finally, multiple logistic regressions were conducted to examine whether one of the three questions (AoA, Frequency and Recency) or the answers to the questions, could reliably predict either a known or ToT judgement. Due to an imbalance between the total number of known and ToT judgements, the ToT judgements were randomly duplicated to match the number of known judgements. This upsampling was conducted to help remove any biases that can exist towards the majority class when conducting logistic regressions on imbalanced data. Furthermore, due to the presence of multiple variables, as well as improved interpretability, it was decided to present the odds ratios (*OR*) in the results. For the *OR*s, values > 1 represent an increased likelihood of a ToT as the predictor increases, while values < 1 represent a decrease in the likelihood of a ToT as the predictor increases. Failure to reject the null hypothesis was defined as an OR of 1. Furthermore, we failed to reject the null hypothesis if the value of 1 fell within the 95% confidence interval of the OR within each predictor. All statistical analyses were performed in Python using the Pingouin statistics library^39^.

## Data Availability

Raw data, experimental files and analyses scripts for this experiment can be found at (https://osf.io/hbaqr/).
